# *AcCHI*, a Chitinase from *Atriplex canescens*, Confers Enhanced Tolerance to Osmotic and Salt Stress in *Glycine max* by Reinforcing Antioxidant and Osmotic Responses

**DOI:** 10.3390/plants15192939

**Published:** 2026-09-25

**Authors:** Jianan Sun, Shishu Luo, Jiaming Zhang, Jiyang Li, Yibo Zhou, Bo Li, Mingmin Zhao, Qiang Lyu

**Affiliations:** 1 Jilin Provincial Key Laboratory of Plant Resource Science and Green Production, Jilin Normal University, Siping 136000, China; 2 College of Horticulture and Plant Protection, Inner Mongolia Agricultural University, Hohhot 010010, China

**Keywords:** chitinase, *Glycine max*, *Atriplex canescens*, abiotic stress, ABA sensitivity, osmolyte accumulation

## Abstract

Soybean is the most crucial oilseed and plant-protein crop worldwide, yet its yield is frequently threatened by salinity and drought stress. In this study, the chitinase gene *AcCHI* was cloned from the highly stress-tolerant plant *Atriplex canescens* and heterologously overexpressed in soybean to generate stable T_3_ homozygous transgenic lines. Wild-type and transgenic plants were subjected to water-withholding drought treatment, 20% PEG6000-induced osmotic stress, and 150 mM NaCl salt stress. After drought treatment, the survival rate of transgenic lines reached 2.66–2.69 times relative to wild-type plants. Under osmotic and salt stress conditions, *AcCHI*-overexpressing lines exhibited better-maintained photosynthetic performance, higher proline accumulation, and lower malondialdehyde (MDA) levels compared with wild-type controls. Enhanced antioxidant enzyme activities and weakened histochemical ROS staining were also observed in transgenic leaves. Stress-responsive genes associated with DREB, RD22, and SOS pathways showed stronger stress-inducible expression in transgenic plants. Our results show that heterologous *AcCHI* expression is associated with improved osmotic- and salt-stress tolerance in soybean seedlings under laboratory conditions. This study provides new observational insights into chitinase-linked abiotic-stress responses and offers candidate genetic resources for breeding stress-tolerant soybean varieties.

## 1. Introduction

Soybean is an important oilseed and protein crop, with its seeds containing 35% to 40% protein, which makes it a rich source of plant protein. Northeast China, a major soybean-producing region, contributes about 41% of the country’s total soybean output [1]. However, in recent years, environmental stresses have adversely impacted soybean growth and yield, with factors such as salinity, drought, and low temperatures severely affecting soybean development.

Against the backdrop of increasingly severe global climate change, the problems of soil salinization and desertification are becoming more acute worldwide. Current research focuses on identifying key genetic resources and elucidating the molecular mechanisms underlying soybean stress tolerance, with the aim of overcoming bottlenecks in enhancing soybean stress tolerance [2,3]. Drought and soil salinization, exacerbated by climate change, are the primary abiotic stressors limiting soybean yields. These stressors often occur simultaneously, with their core damage mechanisms involving osmotic, ionic, and oxidative damage that inhibit plant growth [3]. Soil water deficit directly causes osmotic stress, which limits water uptake by the root system and leads to cellular dehydration. In regions affected by soil salinization, drought conditions further concentrate salts in the soil solution, forcing plants to absorb excessive harmful ions like Na^+^ and Cl^−^ and disrupting intracellular ion homeostasis. The synergistic effect of osmotic stress and ionic toxicity induces a burst of reactive oxygen species (ROS) accumulation, which in turn triggers membrane lipid peroxidation and irreversible damage to photosynthetic machinery [4,5]. Substantial progress has been made in dissecting soybean abiotic-stress response pathways, and multiple stress-related genes participating in ion homeostasis, antioxidant defense and ABA-dependent signaling have been identified [6,7,8]. Even so, our understanding remains incomplete. Most existing functional studies concentrate on soybean endogenous genes from glycophytic backgrounds, while the potential of stress-adapted genes derived from extremophile halophytes remains under-explored for soybean genetic improvement, creating an important research gap for further investigation.

*Atriplex canescens* thrives under drought, saline-alkali, cold, heavy metals, and even high-temperature stress conditions [9,10]. It has been confirmed to be a salt-excreting halophyte, and its reactive oxygen species (ROS) scavenging system is significantly activated under salt stress [11,12]. The heterologous expression of the *AcCHI* and *AcF3H* genes in Arabidopsis confirmed that both genes promote the accumulation of flavonoids to maintain the homeostasis of reactive oxygen species [13]. Specifically, *AcF3H* confers significant dual tolerance to salt and drought in Arabidopsis [14]. Additionally, high-level expression of the plasma membrane intrinsic protein *AcPIP2* from *Atriplex canescens* in *Arabidopsis thaliana* was found to enhance plant growth rate and abiotic stress tolerance [15]. Furthermore, the expression of a heavy metal-associated protein (AcHMA1) from *Atriplex canescens* in *Saccharomyces cerevisiae* conferred tolerance to iron and other abiotic stresses [16]. Notably, an ErbB-3-binding protein gene *AcEBP1*, also known as the *proliferation-associated 2G4* gene (PA2G4s) belonging to the M24 superfamily, was obtained from the saltbush *Atriplex canescens.* It may function by negatively regulating cell growth and stress responses in *Arabidopsis thaliana* [17]. These results indicate that the *Atriplex canescens* genome contains a rich source of stress-resistance genes, providing genetic resources for improving soybean stress tolerance.

Pathogenesis-related (PR) proteins are synthesized in plants in response to biotic and abiotic stresses, with their expression regulated by signaling molecules like salicylic acid and jasmonic acid. These proteins are crucial for enhancing plant resistance to external threats. To date, a total of 19 pathogenesis-related (PR) protein families (PR-1 to PR-19) have been identified, with PR-3 consisting of chitinases [18]. Chitinases belong to the glycoside hydrolases (GH) that catalyze the hydrolysis of natural polymers such as chitin, chitosan, chitosan oligosaccharides, and peptidoglycans [19]. Chitin, a major component of fungal cell walls, prompts plants to secrete chitinases during fungal infections, thereby resisting invasion and activating defense signaling pathways, while chitinases play a role in protecting plants against fungal pathogens [20,21,22]. Recent research has revealed that chitinases not only help plants resist biotic stress but also play a significant role in abiotic stress conditions. For example, chitinase gene *Chi11* enhances tomato tolerance to salt and drought stress [23]. In addition, overexpression of *HvChi22* in *Arabidopsis thaliana* enhances drought tolerance by boosting oxidative stress-responsive enzyme activity, thus reducing MDA accumulation [24]. In maize, *ZmChi7* confers salt stress tolerance by regulating ROS homeostasis [25]. Further studies have confirmed that chitinases not only hydrolyze the cell walls of pathogens to defend plants against fungal invasion but also significantly enhance plant response to abiotic stress by activating stress signaling pathways within the plant [26,27]. Nevertheless, most functionally characterized abiotic-stress-related chitinases are derived from glycophytic crop species. Whether chitinase genes from extremophile halophytes can improve stress tolerance in soybean remains largely unexplored.

Accordingly, we hypothesized that heterologous expression of *AcCHI* isolated from *Atriplex canescens* may improve soybean performance under drought, osmotic, and salt stress, possibly via modulating plant antioxidant and osmotic-related responses. To test this hypothesis, we introduced *AcCHI* into soybean and generated stable T_3_ homozygous overexpression lines. We subjected wild-type and transgenic soybean plants to water-withholding drought, PEG-simulated osmotic stress, and NaCl-imposed salt stress, and evaluated plant phenotypes, photosynthetic performance, physiological-biochemical indices, as well as stress-responsive gene expression profiles. This work aims to explore the potential value of the halophyte-origin *AcCHI* as a genetic resource for improving soybean abiotic-stress adaptation.

## 2. Results

### 2.1. Isolation and Characterization of AcCHI

A partial EST sequence of *AcCHI* (GenBank accession: JZ536011) was retrieved from the *Atriplex canescens* cDNA library [28]. The full-length cDNA encoding the putative CHI protein was subsequently amplified using the RACE method (Figure S1). To characterize the AcCHI protein, the physicochemical properties were predicted using the online ProtParam tool. The results show that the *AcCHI*-encoded protein has the molecular formula C_1277_H_1919_N_355_O_394_S_21_ and consists of 278 amino acids. It has a theoretical molecular mass of approximately 29 kDa and a theoretical isoelectric point of 8.70. Furthermore, it exhibits an aliphatic index of 53.45 and a grand average of hydropathicity (GRAVY) of −0.137, suggesting it is a hydrophilic protein.

In this study, we selected CHI protein sequences from *Atriplex canescens*, *Arabidopsis thaliana*, wheat (*Triticum aestivum*), soybean (*Glycine max*), rice (*Oryza sativa*), and alfalfa (*Medicago truncatula*) to analyze the evolutionary relationship between AcCHI from *Atriplex canescens* and CHI family-encoding proteins in common crops (Figure 1). Phylogenetic analysis showed that AcCHI clustered together with wheat CHI homologs, although this node exhibited low bootstrap support. It next clustered with the proteins encoded by rice *CHI* genes (e.g., LOC_Os04G41680 and LOC_Os04G41620) in the phylogenetic tree. The proteins encoded by the *CHI* genes in soybean and *Medicago truncatula* showed a more distant relationship to AcCHI, while AcCHI is most distantly related to the protein encoded by the *CHI* gene in *Arabidopsis*.

### 2.2. Overexpression of AcCHI Enhances Soybean Tolerance to Drought and 20% PEG6000 Osmotic Stress

To characterize the biological function of AcCHI, we constructed *35S::*AcCHI overexpression soybean lines via *Agrobacterium*-mediated genetic transformation. After PCR identification, purification and screening of T_1_ transgenic soybeans, we advanced the plants to the T_3_ generation to generate homozygous lines, and three lines (named OE1, OE2 and OE3) were chosen for subsequent experiments (Figure S2a). Quantitative Reverse Transcription Polymerase Chain Reaction (qRT-PCR) analysis revealed that no expression of the *AcCHI* gene was detected in wild-type “SN9” plants, whereas *AcCHI* exhibited high transcript abundance in independent transgenic lines (*p* < 0.01, Figure S2b).

Wild-type (WT) and three transgenic lines were planted in flower pots. After 14 days of normal growth, all lines exhibited consistent growth patterns, with no significant differences in plant height, leaf relative water content (RWC) or growth vigor (Figure 2a–c). However, under the water-withholding treatment (drought) for 7 days, the wild-type plants exhibited severe water loss and wilting, with curled and yellowed leaves and virtually no further growth. In contrast, the transgenic lines remained largely green with only mild leaf drooping (Figure 2a). After 7-day drought treatment, plant growth was inhibited, and the final plant height of drought-stressed seedlings was lower than that of well-watered plants (Figure 2b). The trend in RWC was consistent with the trend in plant height. After 7 days of drought, the RWC of wild-type plants dropped to 63.71%, while that of the transgenic lines remained between 82.7% and 85.98% (Figure 2c). After 7 days of drought treatment followed by rewatering, around 80% of transgenic plants survived, whereas only 30% of WT plants survived (*p* < 0.0001, Figure 2d). This phenotype is in accordance with their significantly lower leaf water-loss rate (*p* < 0.0001, Figure 2e).

In a further assay, wild-type (WT) and three overexpression transgenic lines (OE1–OE3, hereafter referred to as OEs) were subjected to 20% (*w*/*v*) PEG6000 osmotic stress, and phenotypic changes and physiological indices were subsequently characterized. Under normal hydroponic conditions (Mock), no significant differences were noted in growth, plant height, leaf morphology, or vigor between the wild-type and OEs (Figure 3a). However, following the 20% PEG6000 treatment, growth inhibition occurred in all lines. Wild-type plants exhibited severe leaf wilting and drooping by 96 h, whereas the transgenic lines showed only slight leaf curling and maintained a relatively normal growth state (Figure 3a, red arrows). Plant heights in the 20% PEG6000 treatment group were all significantly lower than those in the control group, but the plant heights of the transgenic lines in that group were significantly higher than those of the wild-type (*p* < 0.0001, Figure 3b). Treatment with 20% PEG6000 resulted in a decrease in chlorophyll content in all lines. WT chlorophyll declined by 29.6%, whereas chlorophyll in transgenic lines only dropped 16–17.6%, retaining markedly higher chlorophyll levels (*p* < 0.0001, Figure 3c)). Under normal hydroponic conditions, all lines maintained low proline (PRO) and malondialdehyde (MDA) levels with no significant differences. After 96 h of 20% PEG6000 stress, PRO and MDA contents sharply increased in all lines. Comparison of the stress-induced increments (treatment − control) between WT and OEs showed that the proline increment in the OE lines reached 5.85–6.10 times that in WT, whereas the MDA increment was only 34.1–36.2% of that in WT, and both differences were extremely significant (*p* < 0.0001, Figure 3d,e).

Under normal growth conditions, WT and OEs showed no significant differences in stomatal conductance (Gs), transpiration rate (Tr), intercellular CO_2_ concentration (Ci), and net photosynthetic rate (Pn, *p* > 0.05, Figure 4a–d). With prolonged osmotic stress duration, Gs, Tr, Ci and Pn gradually decreased in all lines. At 48 h post-stress, Gs, Tr and Pn in WT dropped by 15.8%, 36.1% and 69.4% relative to 0 h, whereas Ci only declined by 3.3% (*p* > 0.05, non-significant). The decreases in Gs and Tr values in the transgenic lines were significantly greater than those in the wild-type, declining by 47.9–55.9% and 74.6–77%, respectively, while Ci decreased significantly by 27.4–33.3% (*p* < 0.05), whereas the decrease in Pn was only 34.4–45.3%, compared with the 69.4% loss in WT. After 96 h of osmotic stress, photosynthetic parameters further declined in all lines, yet transgenic lines retained substantially higher photosynthetic capacity than WT. At this point, WT Gs, Tr and Pn fell by 31.2%, 71.4% and 84.5% relative to 0 h, whereas Ci only dropped by 15.5% and stayed at a relatively high level. The Gs, Tr, and Pn values of the OEs decreased by 66.2–67.8%, 82.6–83.2%, and 49.5–60.4%, respectively, and their Ci values also decreased significantly by 33.6–39.9%. This suggests that under prolonged severe osmotic stress, transgenic lines possess partial stomatal adjustment capability. Even with pronounced stomatal closure, they maintain better photosynthetic performance and a higher net photosynthetic rate.

### 2.3. AcCHI Overexpression Is Associated with Up-Regulation of Stress-Related Genes in Soybean Under PEG-Mediated Osmotic Stress

Considering the consistent phenotypic performance among three independent transgenic lines observed in phenotypic assays, we selected two representative overexpression lines (OE1, OE2) for subsequent qRT-PCR quantification of three core stress-responsive genes (*GmDREB1B*, *GmDREB2*, *GmRD22*) in WT and transgenic leaves under 20% (*w*/*v*) PEG6000 osmotic stress (Figure 5a–c). Transcript levels of these genes were markedly elevated in all lines with prolonged osmotic stress, yet transgenic lines displayed stronger and higher expression peaks than WT. Both *GmDREB1B* and *GmDREB2* reached peak expression 3 h after treatment (Figure 5a,b). At this point, *GmDREB1B* expression levels in the transgenic lines OE1 and OE2 were 2.91-fold and 2.19-fold higher than those in the wild-type, respectively, while *GmDREB2* expression levels were 1.86-fold and 1.66-fold higher than those in the wild-type, respectively (*p* < 0.0001). *GmRD22* is an important downstream functional gene in the ABA-dependent signaling pathway, and its expression level peaked at 6 h (Figure 5c). In the transgenic lines OE1 and OE2, its expression levels were 1.59-fold and 1.54-fold higher than those of the wild-type, respectively (*p* < 0.0001). During the 24-h stress treatment, expression levels in both transgenic lines were higher than those in the wild-type.

### 2.4. Overexpression of AcCHI Enhances Soybean Tolerance to Salt Stress

Under 150 mM NaCl salt stress, WT suffered far more severe stress damage than the OEs. After 3 days of salt treatment, WT leaves displayed obvious yellowing and wilting accompanied by growth arrest, while transgenic lines maintained green leaves with only mild growth retardation and significantly greater plant height than WT (Figure 6a). After 3 days of salt stress, final plant height was 44.7% lower than that of untreated controls. By contrast, overexpression lines exhibited only a 6.8–10.6% reduction in height and maintained significantly greater plant height (*p* < 0.0001, Figure 6b). WT chlorophyll content decreased by 34.6%, compared with a mild 10.4–12.6% reduction in transgenic lines, which maintained higher pigment levels (*p* < 0.0001, Figure 6c). Salt stress markedly elevated leaf PRO and MDA contents in all lines. Comparison of the stress-induced increments (treatment- control) between WT and OEs revealed that proline increment in OEs reached 2.00–2.11 times that in WT, whereas the MDA increment was only 9.53–12.02% of that in WT (*p* < 0.0001, Figure 6d,e).

We further analyzed leaf photosynthetic parameters of WT and overexpression lines under 150 mM NaCl salt stress. With prolonged salt treatment, Gs, Tr, Ci and Pn continuously declined in all lines (Figure 7a–d). At 24 h post-stress, WT Gs, Tr and Pn fell by 17%, 27.2% and 56.6% relative to 0 h, with Ci only reduced by 11.7%. Transgenic lines displayed milder declines in Pn (43.8–44.5%) yet larger reductions in Gs (18.77–23.09%), Tr (46.74–48.28%) and Ci (19–23.02%, *p* < 0.05). At 72 h of salt treatment, all lines exhibited further reduced photosynthetic parameters, whereas transgenic lines retained stronger photosynthetic capacity. Relative to 0 h, WT Gs, Tr and Pn decreased by 27%, 69.1% and 77.1%, and Ci dropped by 19.9%. Under the same treatment, transgenic lines showed greater reductions in Gs (45.7–49.2%), Tr (83.3–84.0%) and Ci (30.4–34.8%), whereas their Pn was less inhibited with a reduction of 55.6–57.2%. Collectively, although transgenic lines exhibited stronger stomatal closure under prolonged severe salt stress, they were able to sustain higher photosynthetic capacity.

### 2.5. AcCHI Overexpression Alters Ion Transport and Stress-Responsive Genes in Soybean Under Salt Stress

The expression profiles of six core stress genes, including three salt-responsive ion transporter genes (*GmCLC1*, *GmNHX1*, *GmSOS1*) and three abiotic stress-related genes (*GmDREB1B*, *GmDREB2*, *GmRD22*), were analyzed under 150 mM NaCl stress (Figure 8a–f). Salt stress markedly induced all six genes, with transgenic lines showing faster, stronger induction and higher expression peaks than WT. For ion transport-related genes, both *GmCLC1* and *GmSOS1* reached their expression peaks 6 h after stress onset. At this time, the *GmCLC1* expression levels in the transgenic lines OE1 and OE2 were 2.62-fold and 2.67-fold higher than those in the wild-type, respectively, and *GmSOS1* expression levels were 2.96-fold and 2.77-fold higher than those of the wild-type, respectively, with all differences being extremely significant (*p* < 0.0001). The expression of *GmNHX1* peaked at 3 h in OE1 and at 6 h in OE2, with expression levels in OE1 and OE2 being 2.72-fold and 1.54-fold higher, respectively, than in the wild-type (*p* < 0.0001). The *GmDREB1B* transcription factor gene reached its peak expression 6 h after stress, with expression levels in the transgenic lines being 3.07-fold and 2.46-fold higher than those in the wild-type, respectively (*p* < 0.0001). *GmDREB2* and *GmRD22* showed peak expression at 3 h and 6 h, respectively. At these peak time-points, *GmDREB2* was 1.85-fold (OE1) and 2.56-fold (OE2) higher than WT, while *GmRD22* was 1.56-fold (OE1) and 3.7-fold (OE2) higher (*p* < 0.0001). At 24 h after stress, although the expression levels of all genes had decreased, those in the transgenic lines remained significantly higher than those in the wild-type (Figure 8a–f).

### 2.6. Antioxidant Enzyme Activities in Soybean Under 20% PEG-Mediated Osmotic Stress

To explore the physiological basis for enhanced stress-tolerance phenotypes, we assayed the activities of key antioxidant enzymes, since ROS over-accumulation represents an important indicator of stress-induced cellular injury. The peroxidase (POD), catalase (CAT), and superoxide dismutase (SOD) activities increased progressively in all lines with prolonged osmotic stress, yet transgenic lines exhibited markedly greater induction of antioxidant enzymes than WT (Figure 9a–c). Compared with the values at 0 d, POD, CAT and SOD activities in WT at 1 d post-treatment increased by 139.5%, 29.8% and 23.5%, whereas the corresponding increases in the transgenic lines reached 182.5–260.6%, 40.1–45.4% and 78.3–121.8%, respectively (*p* < 0.0001). At 2 d post-treatment, all enzyme activities were further elevated relative to 0 d, with increases of 289.6%, 49.1% and 97.9% in WT versus 411.7–482.1%, 68.0–73.9% and 182.3–213.9% in the transgenic lines (*p* < 0.0001). Collectively, transgenic soybean showed more robust activation of the antioxidant system during the early phase of osmotic stress.

### 2.7. AcCHI Overexpression Is Associated with Reduced ROS Accumulation in Soybean Leaves Under Abiotic Stress

No obvious DAB and NBT staining was observed in WT and transgenic leaves under normal conditions, indicating low and comparable basal ROS levels (Figure 10a,b). Under salt (150 mM NaCl) and osmotic (20% PEG6000) stress, WT leaves displayed intense dark brown DAB staining and deep blue NBT staining, indicating more pronounced accumulation of H_2_O_2_ (hydrogen peroxide) and O_2_.^-^ (superoxide anion). Transgenic leaves only presented sparse stained spots with visibly weaker staining intensity and smaller stained areas compared with WT (Figure 10a,b).

### 2.8. AcCHI Overexpression Enhances ABA-Responsive Inhibition

No obvious differences in germination potential, germination rate and radicle length existed between WT and transgenic lines under normal conditions (*p* > 0.05, Figure 11a–f). 10 μM ABA treatment suppressed seed germination in all lines, with stronger inhibitory effects on transgenic lines. At 4 d, transgenic germination rates (46.4–52.8%) and radicle lengths (0.40–0.43 cm) were markedly lower than WT (63.3%, 0.96 cm, *p*< 0.05, Figure 11a–c). At 7 d, transgenic lines showed germination rates of 61.5–68.6% and radicle lengths of 0.57–0.63 cm, while WT reached 76.5% and 1.76 cm (*p* < 0.05, Figure 11d–f). Under the tested 10 μM exogenous ABA condition, seed germination and radicle elongation of *AcCHI*-overexpressing soybean were more strongly inhibited compared with wild-type.

## 3. Discussion

The present study demonstrates that heterologous expression of *AcCHI*, a chitinase-encoding gene isolated from the xero-halophyte *Atriplex canescens*, is associated with improved soybean performance under water-withholding drought, PEG-simulated osmotic stress, and NaCl-imposed salt stress at the seedling stage. Transgenic lines exhibited better-maintained photosynthetic capacity, higher osmolyte accumulation, lower oxidative damage markers, and enhanced stress-inducible expression of genes linked to DREB-, RD22-, and SOS-associated pathways. Beyond confirming the stress-related functions of plant chitinases in a new crop context, our findings raise two broader questions that merit consideration. First, do halophyte-derived chitinases possess functional properties that distinguish them from glycophytic homologs when deployed in a distant crop genome? Second, does the abiotic stress resilience conferred by *AcCHI* operate through mechanisms that extend beyond simple reinforcement of antioxidant and osmotic responses? With these questions in mind, we discuss our findings in light of current knowledge and propose testable hypotheses for future work.

Plant chitinases have been repeatedly implicated in abiotic stress responses across diverse species. In tomato, *Chi11* overexpression enhanced salt and drought tolerance [23]. In barley, *HvChi22* conferred drought resilience via oxidative enzyme modulation [24], and in maize, *ZmChi7* was shown to regulate ROS homeostasis under salinity [25]. Collectively, these studies support a general principle that elevated chitinase abundance can improve stress performance in glycophytic crops. However, nearly all functionally validated examples derive from crop species themselves or their close relatives. Our work takes this principle a step further, showing that a stress-adapted gene from an extremophile can function effectively in a phylogenetically distant crop genome. This observation aligns with emerging evidence that *Atriplex canescens* harbors multiple genes including *AcF3H* [14], *AcPIP2* [15], and *AcHMA1* [16], all of which confer stress tolerance when heterologously expressed. It also adds chitinase to the repertoire of halophyte-derived genetic resources applicable to crop improvement. The functionality of *AcCHI* in soybean also suggests that downstream stress-response pathways targeted by chitinases are highly conserved. This compatibility enables proteins from distantly related halophytic species to function in crops, which provides valuable insights for future gene mining efforts.

Having established the general feasibility of halophyte-derived chitinase function in soybean, we now turn to a more critical question regarding the nature of the observed phenotypes. An important unresolved issue concerns whether the stress-protective effects reflect a property specific to AcCHI or simply a general consequence of increased chitinase dosage. Soybean possesses its own multigene chitinase family, several members of which are known to be stress- and pathogen-inducible [26]. In principle, overexpression of an endogenous *GmCHI* gene might produce similar effects. Two non-mutually exclusive possibilities could explain our observations. First, AcCHI may possess unique structural or catalytic features such as differential substrate affinity, pH optima, or interaction partners that confer superior stress-alleviating capacity compared with soybean endogenous chitinases. Second, the benefit may derive primarily from quantitative elevation of total chitinase enzyme activity regardless of the gene’s origin, with heterologous AcCHI simply adding to the existing chitinase pool. Distinguishing these possibilities would require parallel overexpression of representative *GmCHI* genes under identical promoter and growth conditions, combined with comparative enzymatic characterization and, ideally, substrate-specificity profiling. These experiments fall outside the scope of the current work but constitute a reasonable direction for future research. Either way, our phenotypic evidence supports the stress-protective role of this halophyte-derived chitinase in soybean and motivates systematic comparisons between chitinase orthologs from extremophile and glycophyte species.

Beyond the question of specificity versus dosage, our gas-exchange data also reveal a noteworthy pattern. The measurements showed that under PEG and NaCl treatments, transgenic soybean exhibited greater relative reductions in Gs compared with WT, while Pn was maintained at relatively higher levels (Figure 4 and Figure 7). This pattern suggests that transgenic plants may have higher intrinsic water-use efficiency (Pn/Gs) under stress. The underlying causes of this response remain unclear. One possible explanation is that non-stomatal limitations to photosynthesis were partially alleviated in transgenic plants. Another possibility is that the accumulation of osmotic substances such as proline (Figure 3d and Figure 6d) may help maintain leaf water status and chloroplast function. Distinguishing between these possibilities would require further validation through approaches such as chlorophyll fluorescence measurements. Overall, these gas-exchange data suggest that the effect of AcCHI overexpression on plant stress tolerance may involve coordination between photosynthetic carbon assimilation and water loss.

The physiological and transcriptional data described above suggest that *AcCHI* overexpression may influence multiple stress-responsive processes. In addition, the enhanced inhibition of seed germination under ABA treatment in *AcCHI*-overexpressing lines (Figure 11), together with the increased transcript abundance of G*mDREB1B, GmDREB2, GmRD22, GmSOS1, GmNHX1* and *GmCLC1* in transgenic plants (Figure 5 and Figure 8), suggests that *AcCHI* may have certain associations with ABA-dependent and ABA-independent stress-responsive networks [29,30]. However, whether these transcriptional changes are directly regulated by AcCHI requires further experimental verification. In the existing literature, PR-3 chitinases are primarily considered to participate in fungal defense [18,31], but recent studies have also found that certain chitinase family members play regulatory roles in abiotic stress responses [32,33]. Our observations are consistent with this trend, showing that heterologous expression of a halophyte-derived *AcCHI* in the glycophytic soybean is associated with improved tolerance to osmotic and salt stress.

Before concluding, we acknowledge several limitations that should be considered when interpreting our findings. First, the absence of an empty-vector control means that transformation-associated effects cannot be completely excluded. However, the consistent phenotypes across three independent transgenic events mitigate this concern, though they do not eliminate it entirely. Second, we have not determined AcCHI protein abundance or chitinase enzymatic activity in transgenic soybeans. Establishing a direct quantitative link between AcCHI protein level and stress-tolerance magnitude would strengthen causal interpretation. Third, all phenotypic assessments were conducted under controlled laboratory conditions at the seedling stage. Multi-year, multi-location field trials on saline or drought-prone soils are indispensable to evaluate whether the observed benefits translate into yield-related agronomic traits [34]. Addressing these limitations in future work, particularly through comparative studies with endogenous *GmCHI* overexpression lines, biochemical characterization of AcCHI catalytic properties, and field-based agronomic evaluation, will be essential to assess the breeding utility of this gene.

## 4. Materials and Methods

### 4.1. Cloning and Bioinformatic Analysis of AcCHI

The partial sequence of AcCHI (GenBank accession: JZ536011) was obtained from Atriplex canescens EST data [28]. Full-length AcCHI cDNA was amplified via 5′ and 3′ RACE using leaf-derived cDNA templates (Vazyme, HiScript-TS 5′/3′ RACE Kit RA101-01, Nanjing, Jiangsu, China). The CDS and amino acid sequences of *AcCHI* are shown in Figure S1. Purified PCR products were ligated into the pMD19-T vector and subjected to Sanger sequencing. The complete open reading frame (ORF) was predicted via NCBI ORF Finder 2.0. Subsequent BLASTP searches and multiple sequence alignment of the deduced amino acid sequence identified conserved chitinase domains, verifying the full-length sequence of *AcCHI*.

The physicochemical properties of the deduced AcCHI protein, including amino acid number and molecular weight, were calculated using the ProtParam tool available on the ExPASy bioinformatics platform [35].

Genomic and protein sequences of Arabidopsis, wheat, soybean, rice and alfalfa were retrieved from Phytozome v14 (https://phytozome.jgi.doe.gov/). Multiple sequence alignment was performed using ClustalW implemented in MEGA 12.0, and a phylogenetic tree was built via the neighbor-joining (NJ) algorithm with 1000 bootstrap replicates to evaluate tree topological reliability [36].

### 4.2. Construction of Overexpression Vector and Soybean Genetic Transformation

The full-length coding sequence (CDS) of *AcCHI* was amplified and inserted into the binary vector pTF101 via *XbaI* and *BamHI* restriction sites. This vector carries the CaMV 35S constitutive promoter and the bar herbicide resistance gene for plant transformant screening. All primer sequences used for vector construction are provided in Supplementary Table S1. The recombinant *pTF101-35S::AcCHI* plasmid was introduced into Agrobacterium tumefaciens strain EHA101 using the freeze–thaw method.

Soybean cultivar Shennong No. 9 (SN9) was subjected to genetic transformation via the cotyledon-node method mediated by engineered *Agrobacterium* [37]. Regenerated T0 seedlings were obtained after 2–3 months of tissue culture, and positive transformants were preliminarily screened on medium supplemented with 5 mg/L glufosinate based on the expression of the *Bar* marker gene. The resistant T_0_ plants were continuously self-pollinated, and the progenies were screened generation by generation. At each generation, stable inheritance of the transgene was verified by PCR amplification of the *AcCHI*-specific fragment from genomic DNA, in combination with glufosinate resistance conferred by the bar marker gene. Finally, three independent homozygous T_3_ overexpression lines (OE1, OE2, and OE3) were selected for subsequent phenotypic and molecular assays.

### 4.3. Molecular Identification of Transgenic Soybean Lines

Transgenic soybean lines overexpressing *AcCHI* and wild-type (WT) soybean cultivar Shennong No. 9 were used as experimental materials. Fresh leaves were harvested from 2–3-week-old soybean seedlings, immediately frozen in liquid nitrogen, and stored at −80 °C for subsequent experiments. Soybean leaf genomic DNA was extracted using the CTAB method [38]. The purity and concentration of extracted DNA were determined via the absorbance values at 260 nm and 280 nm detected by an ultramicro spectrophotometer, and the A_260_/A_280_ ratio was calculated to evaluate DNA quality.

The genomic DNA was used as the template for PCR amplification of the exogenous *AcCHI* gene with specific primers (Supplementary Table S2) [39]. The 20 μL PCR reaction system contained 10 μL of 2× Taq PCR MasterMix, 0.8 μL of forward primer (10 μmol/L), 0.8 μL of reverse primer (10 μmol/L), 1 μL of template DNA (50 ng/μL), and 7.4 μL of ddH_2_O. The PCR amplification procedure was set as follows: initial pre-denaturation at 94 °C for 5 min, 35 cycles of denaturation at 94 °C for 30 s, annealing at 58 °C for 30 s, and extension at 72 °C for 1 min, followed by a final extension at 72 °C for 10 min. Amplified products were temporarily stored at 4 °C after the reaction.

### 4.4. Plant Materials and Stress Treatments

Mature seeds of wild-type (WT) soybean cultivar Shennong No. 9 and *AcCHI*-overexpressing transgenic lines were surface-sterilized sequentially with 75% ethanol for 30 s and 10% sodium hypochlorite for 15 min, followed by 5–6 rinses with sterile distilled water [40]. Sterilized seeds were divided into two culture systems: soil culture for water-withholding treatment and hydroponic culture for polyethylene glycol 6000 (PEG6000) and NaCl stress treatments. All seedlings were cultivated in a controlled environment growth chamber (DRX-600D-LED, Ningbo Kesheng Experimental Instrument Co., Ltd., Ningbo, China) under a 16 h light/8 h dark photoperiod. Day/night air temperatures were set at 25 °C and 22 °C, respectively, and relative humidity was maintained at 80% throughout the experiment. Illumination was provided by full-spectrum LED modules, delivering an incident photosynthetic photon flux density (400–700 nm) of 150 ± 10 μmol·m^−2^·s^−1^ at the top of the plant canopy. For each genotype-treatment combination, at least three independent biological replicates were included, and each biological replicate contained pooled samples from multiple uniformly grown seedlings. Plants were randomly assigned to different treatment groups and positions inside the growth chamber, and pot positions were re-randomized every two days to reduce positional bias.

For the soil culture system, uniform seeds were sown in pots of 6.8 cm × 6.8 cm × 6.5 cm (length × width × height) filled with equal volumes of soil substrate. Before the treatments, all pots were uniformly irrigated and watered regularly during the 14-day pre-treatment cultivation period. The simulated control group received regular watering throughout the entire experiment, while the experimental group was subjected to a 7-day drought treatment by withholding water. After 7 days of drought treatment followed by rewatering, plant survival rate, leaf relative water content, and detached leaf water loss rate were determined according to standard abiotic stress phenotyping protocols [41]. For the hydroponic culture system, sterilized seeds were germinated and cultivated in Hoagland medium for 14 days. Seedlings were assigned to three different culture conditions. The control group was maintained in standard Hoagland solution without additional stress. The osmotic stress group was treated with 1/2 Hoagland’s nutrient solution containing 20% (*w*/*v*) PEG6000 for 96 h, and the salt stress group was treated with 1/2 Hoagland’s nutrient solution containing 150 mM NaCl for 3 days. After the corresponding treatment durations, leaf samples were harvested from all groups for phenotypic observation and subsequent physiological and molecular analyses [42].

### 4.5. Measurement of Photosynthesis-Related Parameters

The topmost trifoliate leaves from each treatment group were selected for the measurement. The net photosynthesis (Pn), transpiration rate (Tr), stomatal conductance (Gs), and intercellular CO_2_ concentration (Ci) were measured using the LI-COR 6800 portable photosynthesis system (LI-COR Biosciences, Lincoln, NE, USA) between 9:00 a.m. and 12:30 p.m. local time with a photosynthetic photon flux density of 800 μmol·m^−2^·s^−1^, CO_2_ concentration at 395–400 μmol·mol^−1^, and 40% leaf chamber air humidity at the ambient air temperature of 25 °C. The average of three measurements taken from the same position on the top trifoliate leaves of each selected plant was used.

Chlorophyll contents of the middle leaflet of the trifoliate leaves were measured using a chlorophyll concentration meter (MC 100, Apogee, Logan, UT, USA). Measurements were performed at appropriate time-points throughout the stress treatment, as indicated in the corresponding figure legends [41].

### 4.6. Determination of MDA and Proline Contents

MDA and proline contents were quantified in wild-type and transgenic soybean fresh leaf tissues under 150 mM NaCl and 20% PEG6000 stress. Seedling samples were harvested at designated stress treatment days for physiological detection.

MDA and PRO contents were determined according to previously published protocols [43,44,45] using commercial assay kits (catalog numbers A003-1-2 and A107-1-1; Nanjing Jiancheng Bioengineering Institute, Nanjing, China).

All absorbance readings were detected with a multifunctional microplate reader. Three independent biological replicates were set for each soybean line.

### 4.7. Determination of SOD, POD and CAT Activities

Soybean leaf samples were collected at different time points during 20% (*w*/*v*) PEG6000 osmotic stress treatments. The third fully expanded functional leaf at the shoot apex was harvested, immediately frozen in liquid nitrogen, and stored at −80 °C for subsequent enzyme extraction. Briefly, 0.5 g of fresh leaf tissue was homogenized fully on ice with 5 mL of pre-cooled extraction buffer (0.1 mol/L phosphate buffer, pH 7.8, supplemented with 0.1 mmol/L EDTA and 1% polyvinylpyrrolidone). The homogenate was centrifuged at 12,000× g for 20 min at 4 °C, and the resulting supernatant was collected as the crude enzyme extract.

The activities of SOD, POD, and CAT were determined using commercial assay kits (catalog numbers A001-3, A084-3-1, and A007-1-1; Nanjing Jiancheng Bioengineering Institute, Nanjing, China) according to the manufacturer’s protocols. All assays were performed with three independent biological replicates.

### 4.8. Histochemical Staining of DAB and NBT

In situ accumulation of H_2_O_2_ and O_2_^−^ in soybean leaves was detected via histochemical staining using 3,3′-diaminobenzidine (DAB) and nitroblue tetrazolium (NBT), respectively. For DAB staining, fresh soybean leaves were immersed in 1 mg·mL^−1^ DAB solution (pH 3.8, containing 10 mmol·L^−1^ Na_2_HPO_4_ and 0.05% Tween-20). After 30 min of vacuum infiltration, the samples were incubated at 25 °C in the dark for approximately 8 h. Subsequently, leaf samples were decolorized in a boiling ethanol–glacial acetic acid mixture (3:1, *v*/*v*) to completely remove chlorophyll pigments. After rinsing with distilled water, the leaf staining phenotypes were observed and photographed under a stereomicroscope.

For NBT staining, consistent leaf samples were submerged in 0.5 mg·mL^−1^ NBT solution prepared with 50 mmol·L^−1^ phosphate buffer (pH 7.8). Following 30 min of vacuum infiltration, all samples from different genotypes were incubated together in the same batch at 25 °C in the dark for approximately 8 h. Chlorophyll was removed by boiling the leaves in anhydrous ethanol, and the samples were rinsed with distilled water. Staining results were finally observed and recorded using a stereomicroscope [46,47].

For histochemical staining of DAB and NBT, three independent biological replicates were performed for each genotype and treatment. Consistent mature functional leaves at the same leaf position of seedlings were collected at the specified time point after stress treatment. All stained samples were photographed under uniform light and background conditions with a fixed camera parameter setting.

### 4.9. RNA Extraction and Quantitative Reverse Transcription PCR Analysis

Total RNA was extracted using FreeZol Reagent (Cat. No. R711-01, Vazyme Biotech Co., Ltd., Nanjing, China) according to the manufacturer’s instructions. First-strand cDNA was synthesized using the HiScript III 1st Strand cDNA Synthesis Kit (+gDNA wiper) (Vazyme, Nanjing, China; Cat. No. R312-01), and the resulting cDNA was diluted 100-fold for subsequent amplification. Quantitative Reverse Transcription PCR was performed using ChamQ Blue Universal SYBR qPCR Master Mix (Cat. No. Q312-02, Vazyme Biotech Co., Ltd., Nanjing, China). Each 20 μL reaction mixture contained 10 μL of 2× ChamQ Blue Universal SYBR qPCR Master Mix, 0.4 μL of each forward and reverse primer (10 μM), 1 μL of cDNA template, and 8.2 μL of RNase-free ddH_2_O. qRT-PCR amplification was performed according to the standard manufacturer’s procedures. The *GmActin* gene was selected as the internal reference. Relative gene expression levels were calculated using the 2^−ΔΔCT^ method, with all analyses performed based on three independent biological replicates [48,49]. Primer sequences for qRT-PCR are provided in Supplementary Table S3.

### 4.10. ABA-Mediated Germination Assay of Soybean Seeds

Wild-type and transgenic soybean seeds were first soaked in warm water at 58 °C and air-dried for two days [50]. Uniformly dried seeds were selected for subsequent germination assays. Sterile filter papers were laid in Petri dishes, with 25 seeds placed evenly in each dish. Seeds were treated with either 20 mL of sterile water (control group) or 20 mL of 10 μM ABA solution (treatment group). All samples were incubated at 25 °C with 60% relative humidity under continuous darkness. Each group contained three biological replicates, with a minimum of 75 seeds in total [51]. A seed was recorded as germinated when the radicle broke through the seed coat and reached a length of at least 2 mm. Each Petri dish was defined as one experimental unit. Germination potential was calculated as the germination percentage at 4 days after treatment, and germination rate was determined as the final germination percentage at 7 days. Radicle length was measured on all germinated seeds within each Petri dish. Germination-related percentage data were analyzed at the Petri-dish level rather than treating individual seeds as independent replicates. Seed germination status was photographed on the 4th and 7th days of cultivation, and the germination potential, germination rate, and radicle length were statistically measured and recorded.

### 4.11. Statistical Analysis

All statistical analyses were performed using GraphPad Prism 10.1.2 software (GraphPad Software, Inc., San Diego, CA, USA) or SPSS 27. Appropriate tests were applied in analyzing these data, meeting assumptions of the statistical methods [52]. Technical replicates from each biological sample were averaged prior to statistical analysis. All analyses were based on three independent biological replicates (*n* = 3). Significant differences were indicated as * *p* < 0.05, ** *p* < 0.01, *** *p* < 0.001, **** *p* < 0.0001. For comparisons among multiple groups, Tukey’s honestly significant difference (HSD) test was applied, and different lowercase letters denote significant differences at *p* < 0.05. All data were expressed as mean ± standard error (SE) of three independent biological replicates.

## 5. Conclusions

In summary, heterologous expression of the halophyte-derived chitinase gene *AcCHI* in soybean is associated with enhanced seedling-stage tolerance to osmotic and salt stress, as evidenced by improved photosynthetic performance, elevated proline accumulation, reduced oxidative damage, enhanced antioxidant enzyme activities, and stronger stress-inducible expression of DREB-, RD22-, and SOS-pathway-related genes. These findings establish *AcCHI* as a candidate genetic resource for soybean stress-tolerance improvement and provide observational evidence linking a halophyte-origin chitinase to abiotic stress resilience in a glycophytic crop. The enhanced ABA responsiveness during seed germination further suggests potential cross-talk between chitinase-associated pathways and ABA signaling, although the molecular basis of this connection requires future investigation. Importantly, the question of whether AcCHI confers stress tolerance through unique structural or catalytic properties, or merely through a general dosage effect shared by endogenous chitinases, remains open and will guide comparative studies with soybean *GmCHI* family members. Field-based agronomic evaluation on saline or drought-prone soils will be a critical next step toward assessing the practical utility of *AcCHI* in soybean breeding programs.

## Figures and Tables

**Figure 1:**
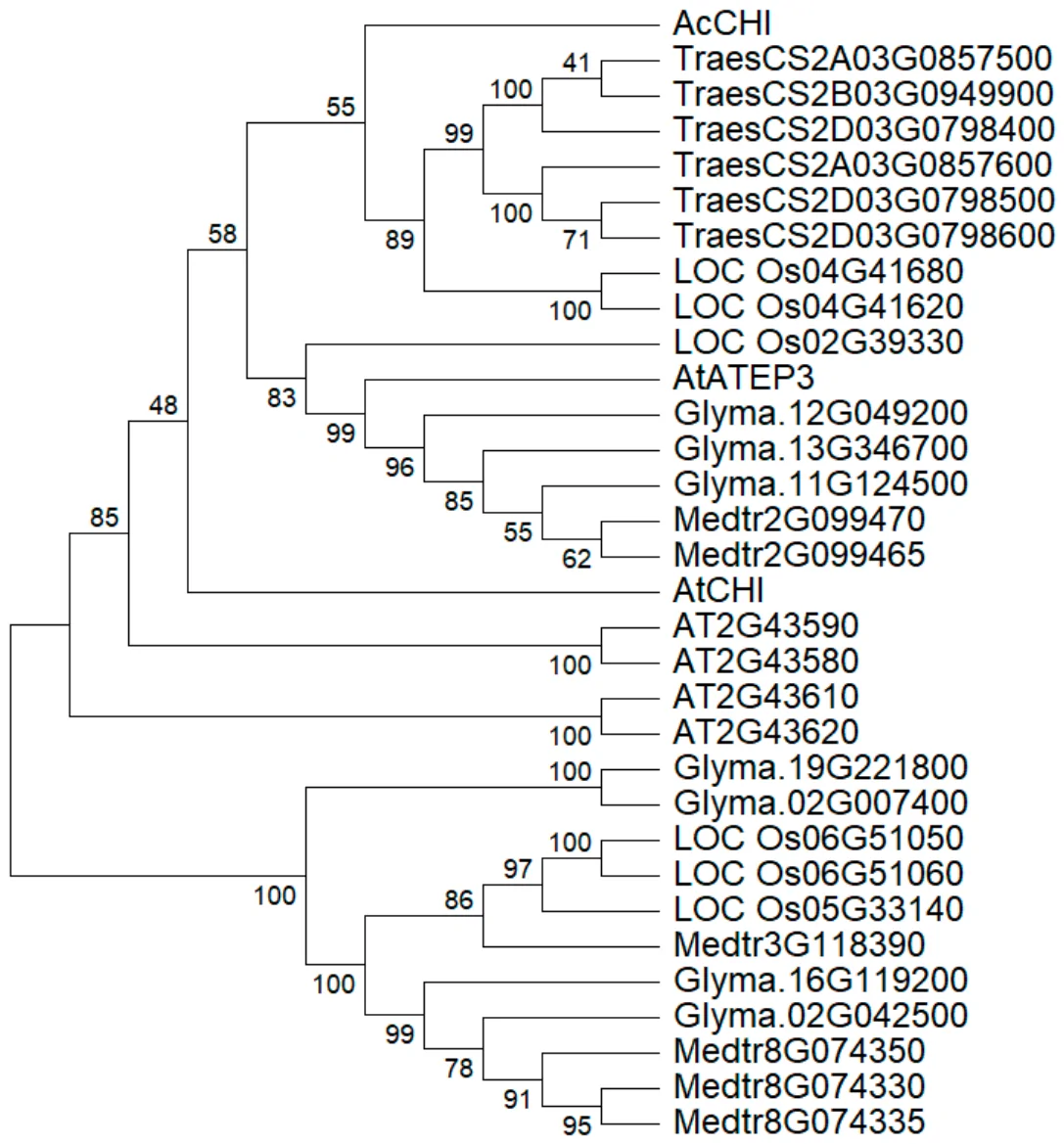
Phylogenetic tree of the protein encoded by the *Atriplex canescens* CHI gene and CHI family proteins from different species. A phylogenetic tree was constructed using the Neighbor-Joining (NJ) method based on the full-length amino acid sequence of the CHI protein. The bootstrap analysis was performed with 1000 repetitions, and the node values represent the bootstrap support values for the branches (only values ≥ 40 are shown). The AcCHI sequence from *Atriplex canescens* obtained in this study is annotated in the figure. The remaining homologous sequences were obtained from the Phytozome database and include CHI family protein sequences from *Triticum aestivum*, *Oryza sativa*, *Arabidopsis thaliana*, *Glycine max*, and *Medicago truncatula*.

**Figure 2:**
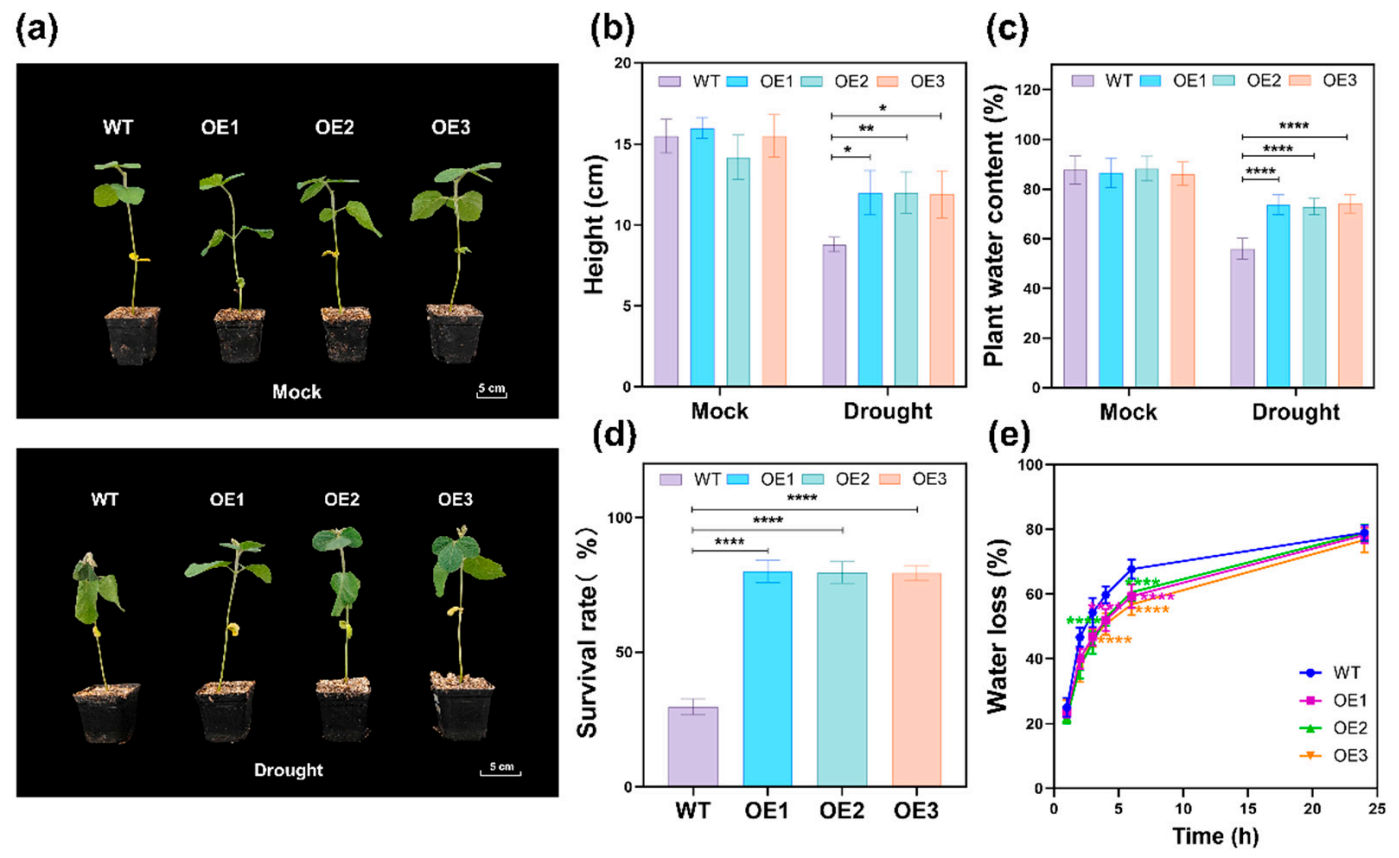
Effects of the water-withholding treatment (drought) on the phenotypes and comprehensive indicators of wild-type (WT) and transgenic soybeans (OE1, OE2, OE3). (**a**) Phenotypes of 14-day-old seedlings after 7 days of well-watered growth or 7-day water withholding; (**b**) Plant height at 7 days under normal growth (Mock) and drought stress conditions; (**c**) Leaf relative water content (RWC) after normal growth and drought stress; (**d**) Survival rate of soybean plants rehydrated following 7-day drought stress; (**e**) Dynamic changes in water loss rates of isolated leaves. Data represent the mean ± standard error (Mean ± SE, *n* = 3) from three independent biological replicates. Each biological replicate comprised nine technical replicates. Asterisks indicate significant differences (Student’s *t*-test), * *p* < 0.05, ** *p* < 0.01, **** *p* < 0.0001. Scale bar = 5 cm.

**Figure 3:**
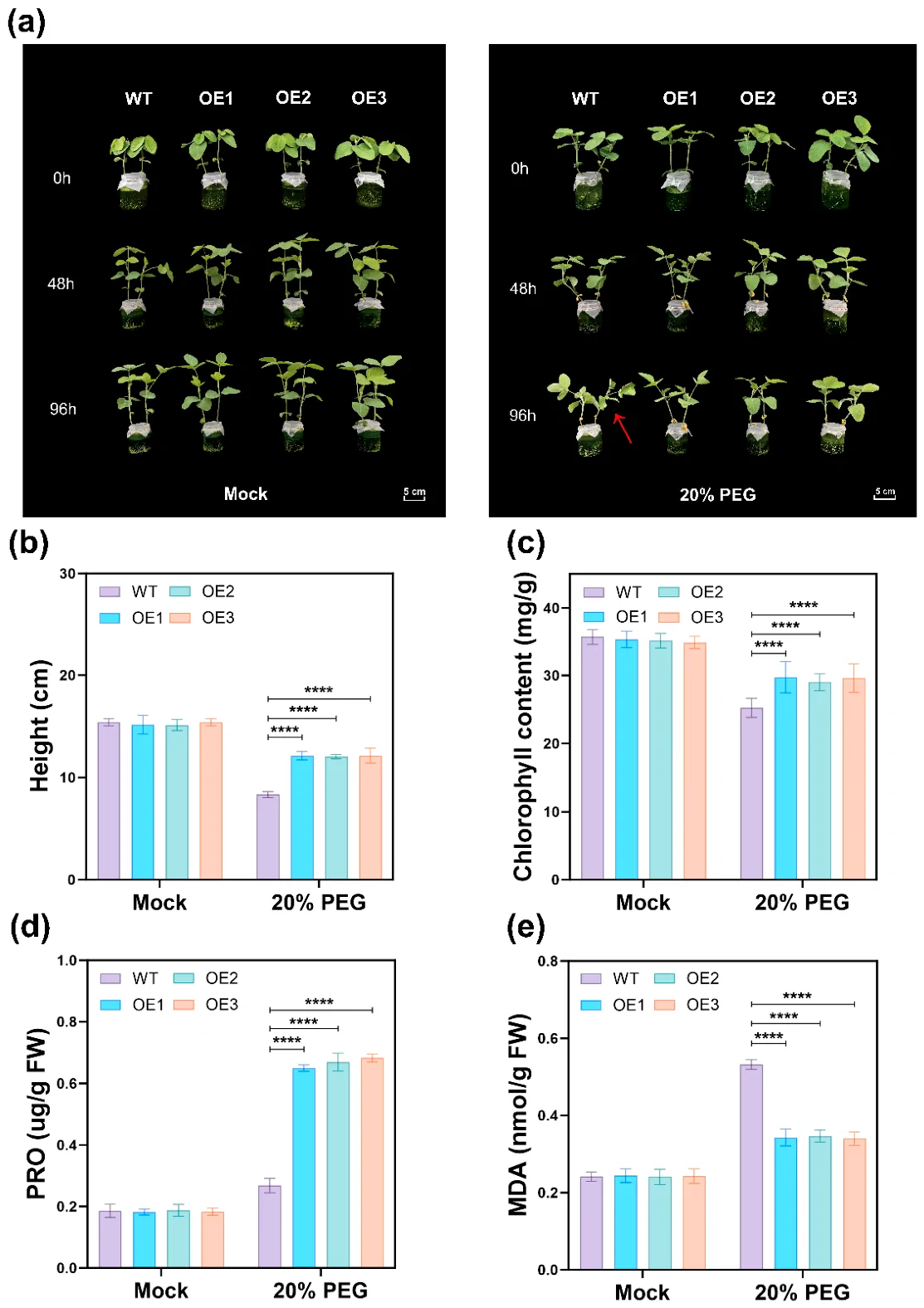
Phenotypic and physiological responses of WT and OE transgenic soybean lines to 20% (*w*/*v*) PEG6000 osmotic stress. (**a**) Phenotypes of 14-day-old soybean seedlings under normal culture or 4-day 20% (*w*/*v*) PEG6000 osmotic stress. Red arrows mark wilted and drooping leaves; (**b**) Plant height 96 h after normal culture and stress treatment; (**c**) Leaf chlorophyll content measured at 96 h under normal and osmotic stress conditions; (**d**) Leaf proline (PRO) contents under normal culture and 96 h osmotic stress treatment; (**e**) Leaf malondialdehyde (MDA) content under normal culture and 96 h osmotic stress treatment. Data represent the mean ± standard error (Mean ± SE, *n* = 3) from three independent biological replicates. Each biological replicate comprised nine technical replicates. Asterisks indicate significant differences (Student’s *t*-test), **** *p* < 0.0001. Scale bar = 5 cm.

**Figure 4:**
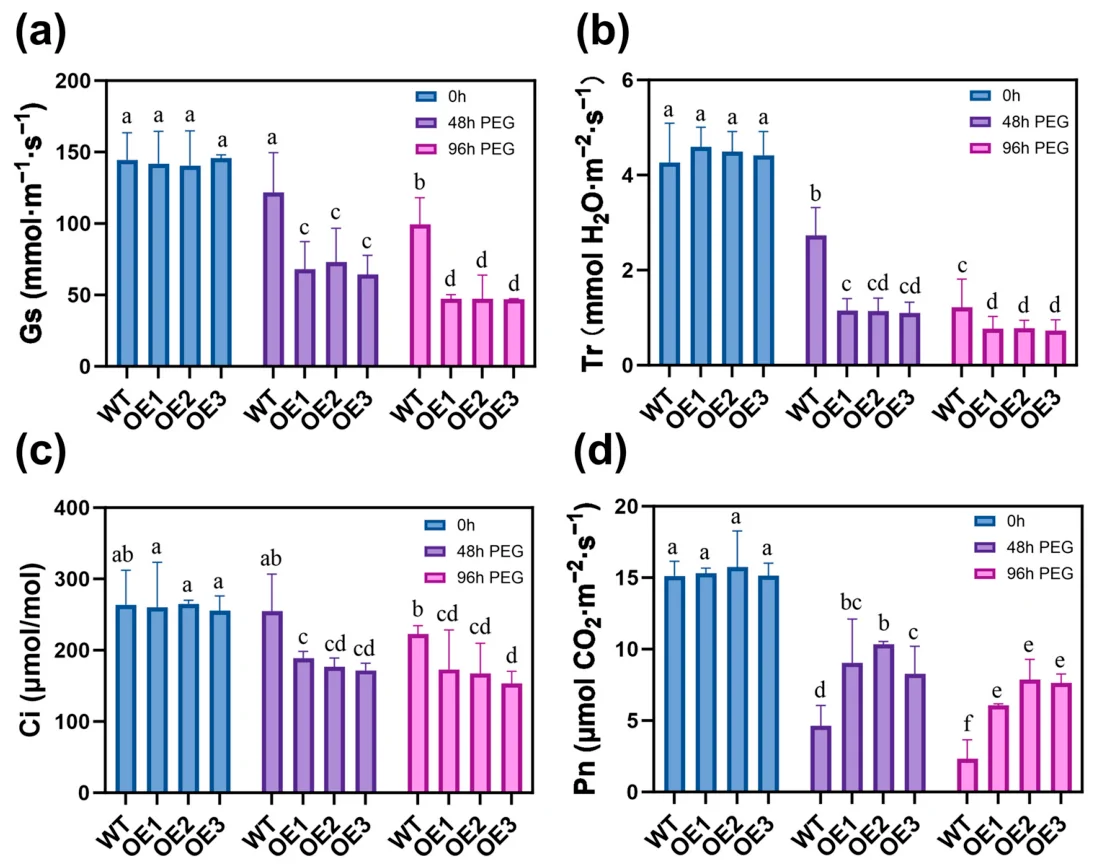
Effects of osmotic stress on leaf photosynthetic parameters of WT and OE transgenic soybean lines. The plants were subjected to osmotic stress with 20% (*w*/*v*) PEG6000 solution. Stomatal conductance (Gs), transpiration rate (Tr), intercellular CO_2_ concentration (Ci), and net photosynthetic rate (Pn) were determined on fully expanded apical functional leaves at 0, 48, and 96 h post-treatment: (**a**) Gs, (**b**) Tr, (**c**) Ci, (**d**) Pn. Tukey’s honestly significant difference (HSD) test was used to compare treatment means following a significant two-way ANOVA. Data represent the mean ± standard error (Mean ± SE, *n* = 3) from three biological replicates, different lowercase letters indicate significant differences among groups (*p* < 0.05). Each biological replicate comprised nine technical replicates.

**Figure 5:**
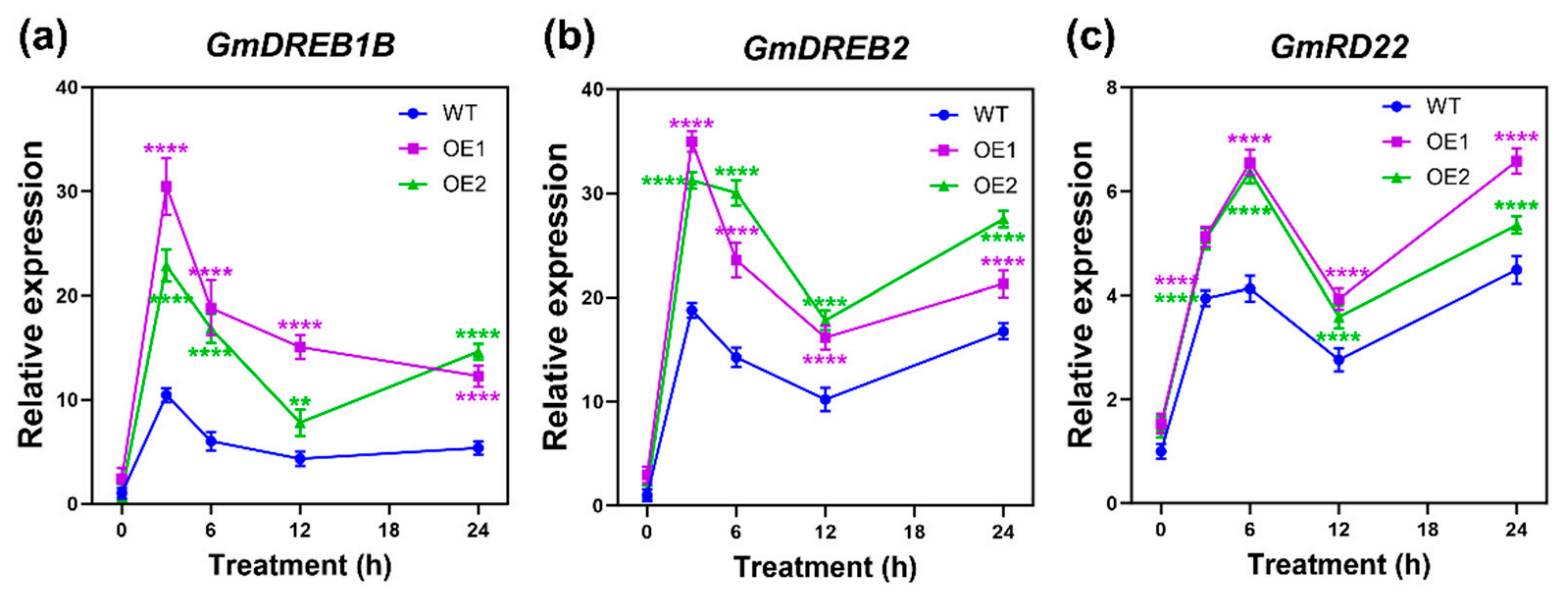
Stress-responsive gene expression profiles of WT and OE transgenic soybean lines under 20% (*w*/*v*) PEG6000 osmotic stress. Relative transcript abundances of (**a**) *GmDREB1B*, (**b**) *GmDREB2* and (**c**) *GmRD22* were quantified by qRT-PCR at 0, 3, 6, 12 and 24 h, normalized to the reference gene *GmActin*. Data are presented as the mean ± standard error (Mean ± SE, *n* = 3) from three independent biological replicates. Asterisks indicate significant differences (Student’s *t*-test), ** *p* < 0.01, **** *p* < 0.0001.

**Figure 6:**
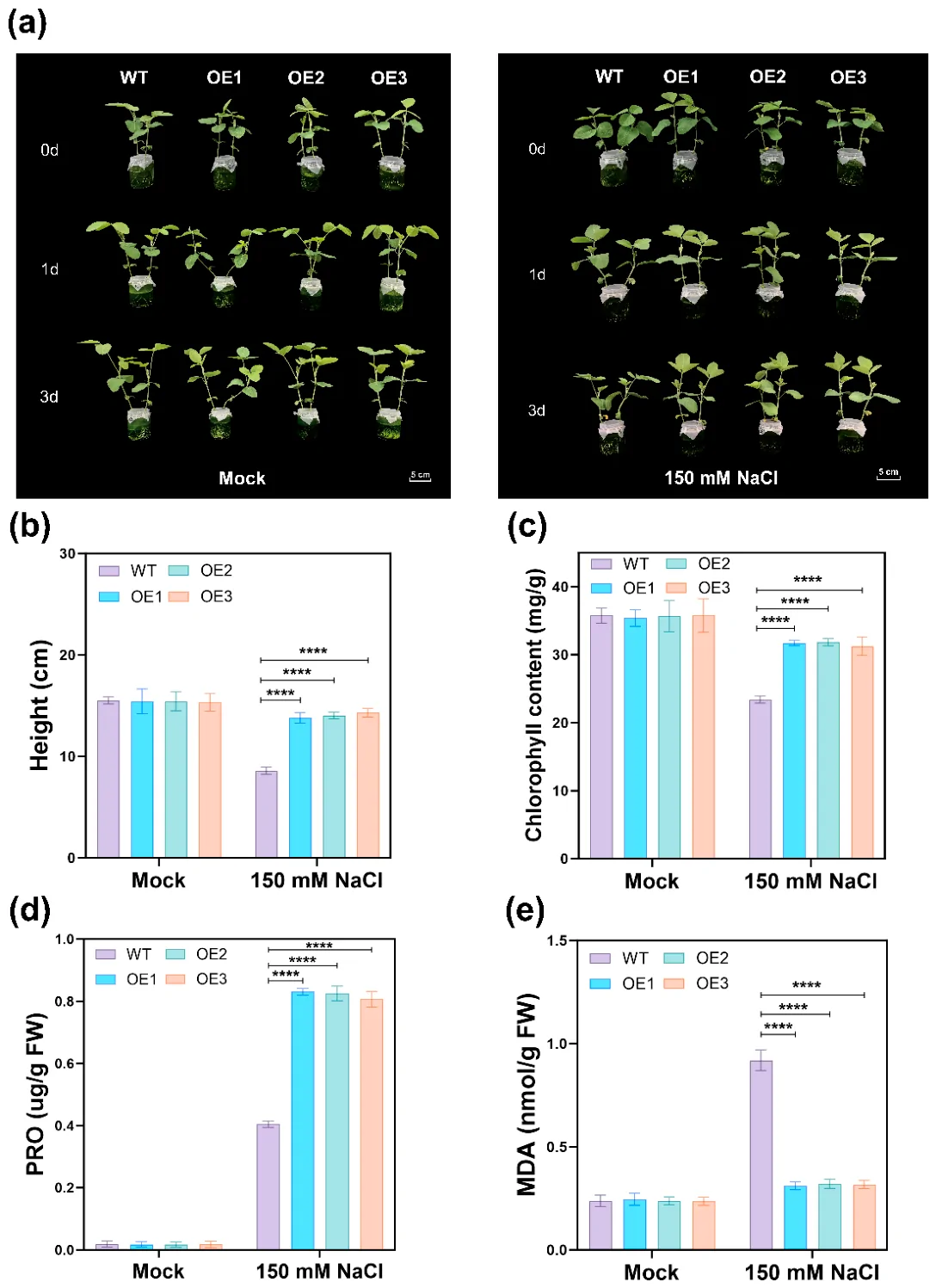
Phenotypic and physiological responses of wild-type (WT) and overexpression soybean lines (OE1–OE3) to 150 mM NaCl salt stress. 14-day-old soybean seedlings were cultured under normal conditions or 150 mM NaCl salt stress for 3 d. The following indicators were measured: (**a**) Seedling phenotypes; (**b**) Plant height; (**c**) Leaf chlorophyll content; (**d**) Leaf proline (PRO) level; (**e**) Leaf malondialdehyde (MDA) level. Data represent the mean ± standard error (Mean ± SE, *n* = 3) from three independent biological replicates. Each biological replicate comprised nine technical replicates. Asterisks indicate significant differences (Student’s *t*-test), **** *p* < 0.0001. Scale bar = 5 cm.

**Figure 7:**
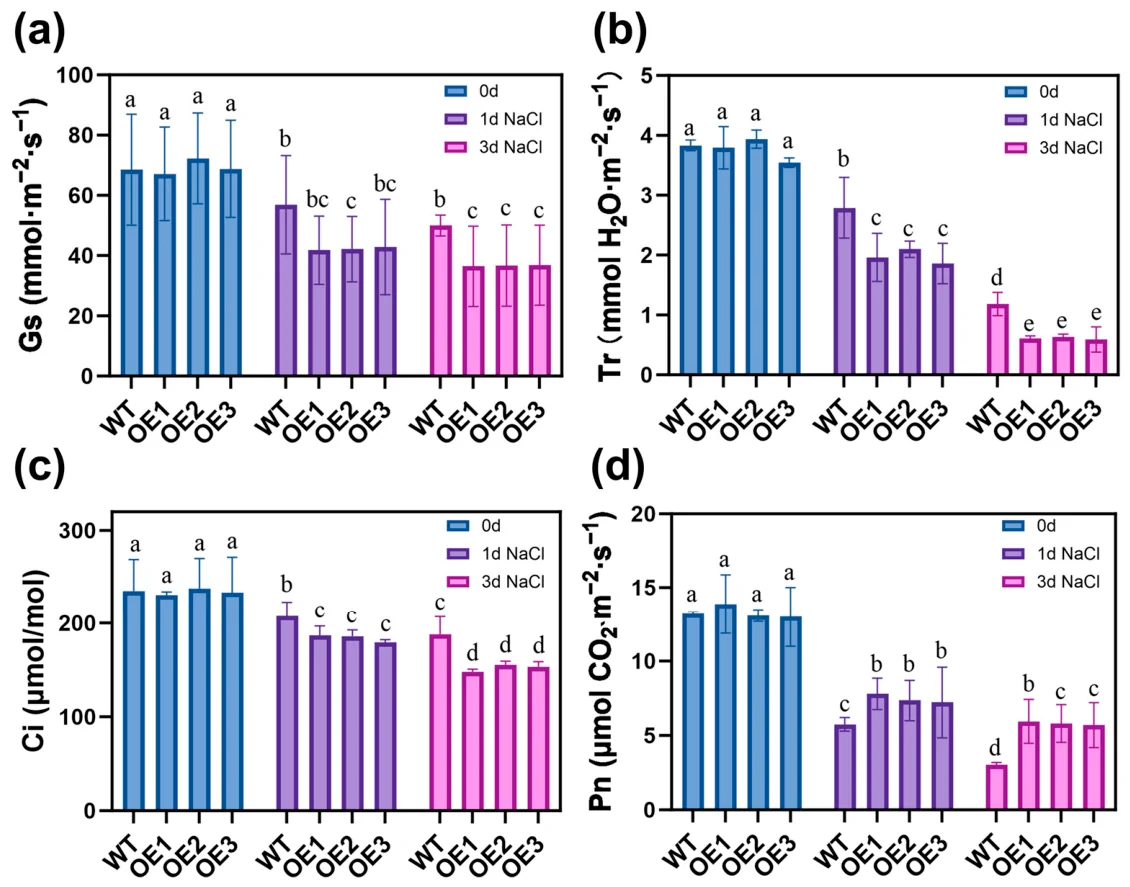
Effects of 150 mM NaCl stress on leaf photosynthetic parameters of WT and OE transgenic soybean lines. Plants were exposed to 150 mM NaCl salt stress. Stomatal conductance (Gs), transpiration rate (Tr), intercellular CO_2_ concentration (Ci), and net photosynthetic rate (Pn) were determined on fully expanded apical functional leaves at 0, 24, and 72 h post-treatment: (**a**) Gs, (**b**) Tr, (**c**) Ci, (**d**) Pn. Tukey’s honestly significant difference (HSD) test was used to compare treatment means following a significant two-way ANOVA. Data represent the mean ± standard error (Mean ± SE, *n* = 3) from three biological replicates, different lowercase letters indicate significant differences among groups (*p* < 0.05). Each biological replicate comprised nine technical replicates.

**Figure 8:**
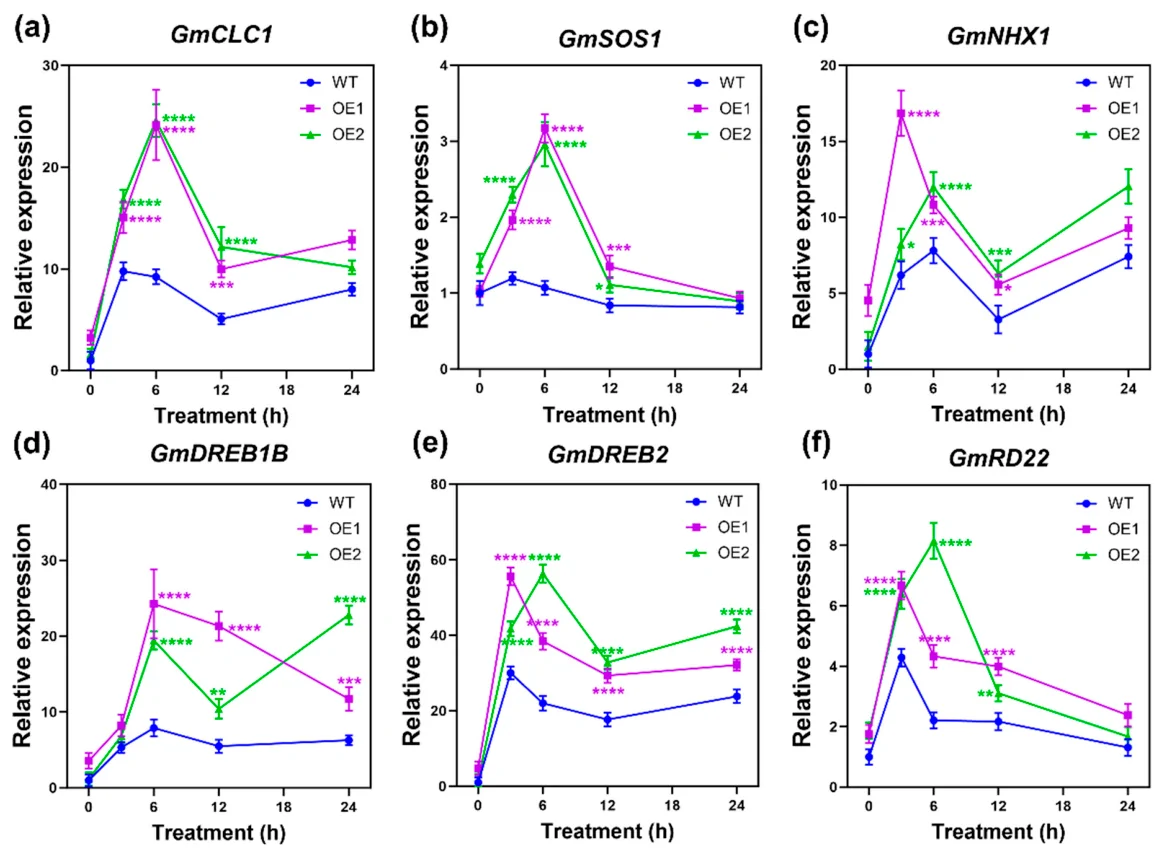
Stress-responsive gene expression profiles of WT and OE transgenic soybean lines under 150 mM NaCl stress. Relative transcript abundances of (**a**) *GmCLC1*, (**b**) *GmSOS1*, (**c**) *GmNHX1*, (**d**) *GmDREB1B*, (**e**) *GmDREB2*, and (**f**) *GmRD22* were quantified by qRT-PCR at 0, 3, 6, 12 and 24 h, normalized to the reference gene *GmActin*. Data are presented as the mean ± standard error (Mean ± SE, *n* = 3) from three independent biological replicates. Asterisks indicate significant differences (Student’s *t*-test), * *p* < 0.05, ** *p* < 0.01, *** *p* < 0.001, **** *p* < 0.0001.

**Figure 9:**
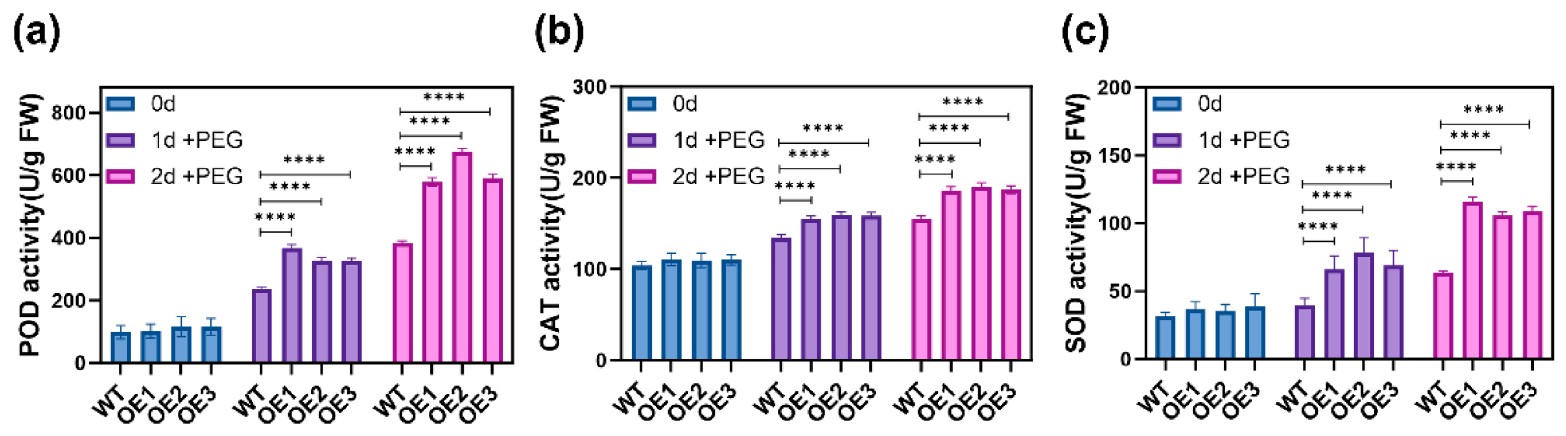
Antioxidant enzyme activities in soybean under 20% (w/v) PEG6000 osmotic stress. Leaves were harvested at 0, 1 and 2 d after 20% (*w*/*v*) PEG6000 treatment to measure (**a**) POD, (**b**) CAT and (**c**) SOD activities. Data represent the mean ± standard error (Mean ± SE, *n* = 3) from three independent biological replicates. Each biological replicate comprised six technical replicates. Asterisks indicate significant differences (Student’s *t*-test), **** *p* < 0.0001.

**Figure 10:**
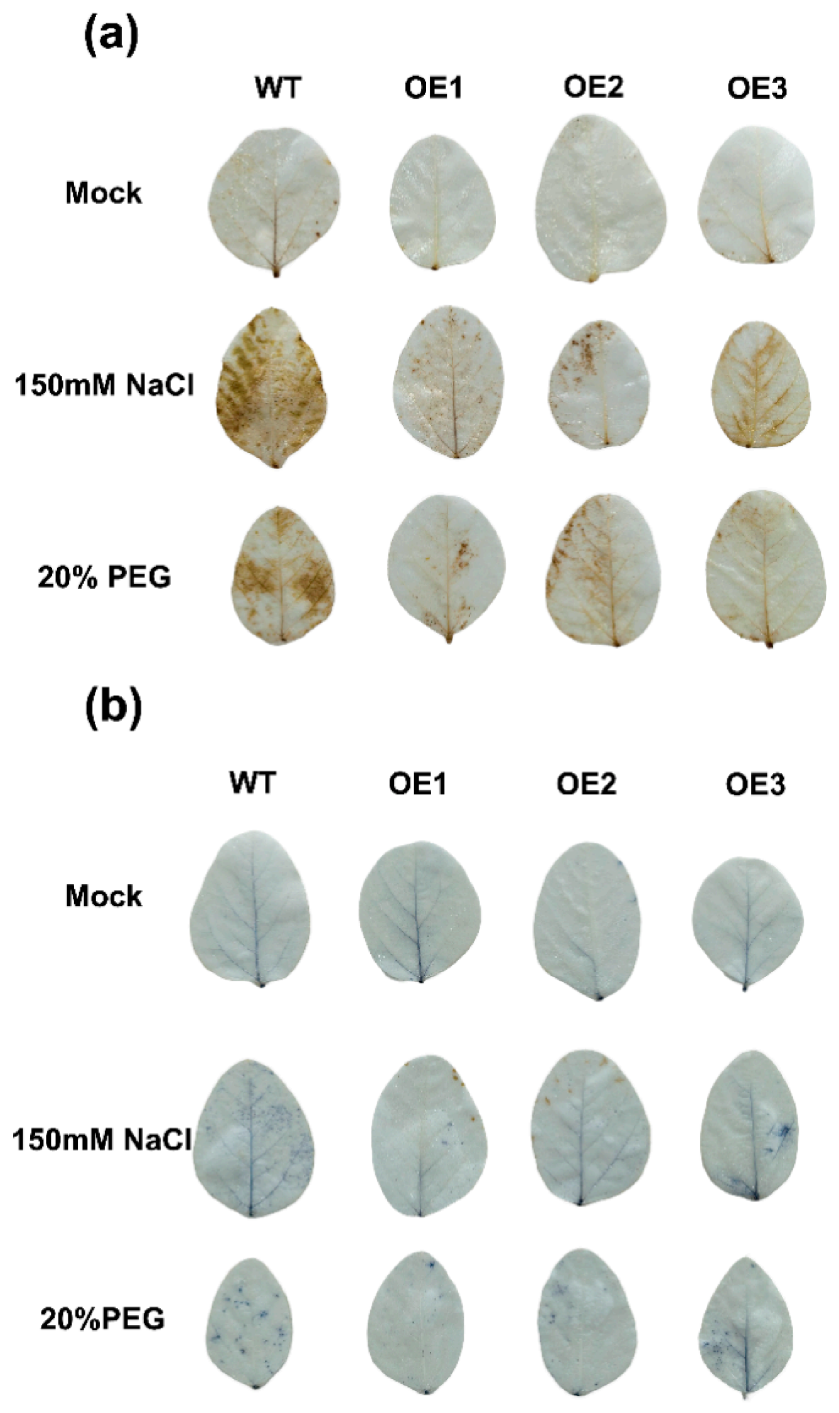
Histochemical staining for ROS in leaves of wild-type and *AcCHI*-overexpressing soybean under abiotic stresses. Leaves were sampled under Mock, 150 mM NaCl and 20% PEG6000 treatments. (**a**) DAB staining for H_2_O_2_ (brown deposits); (**b**) NBT staining for O_2_^−^ (blue deposits).

**Figure 11:**
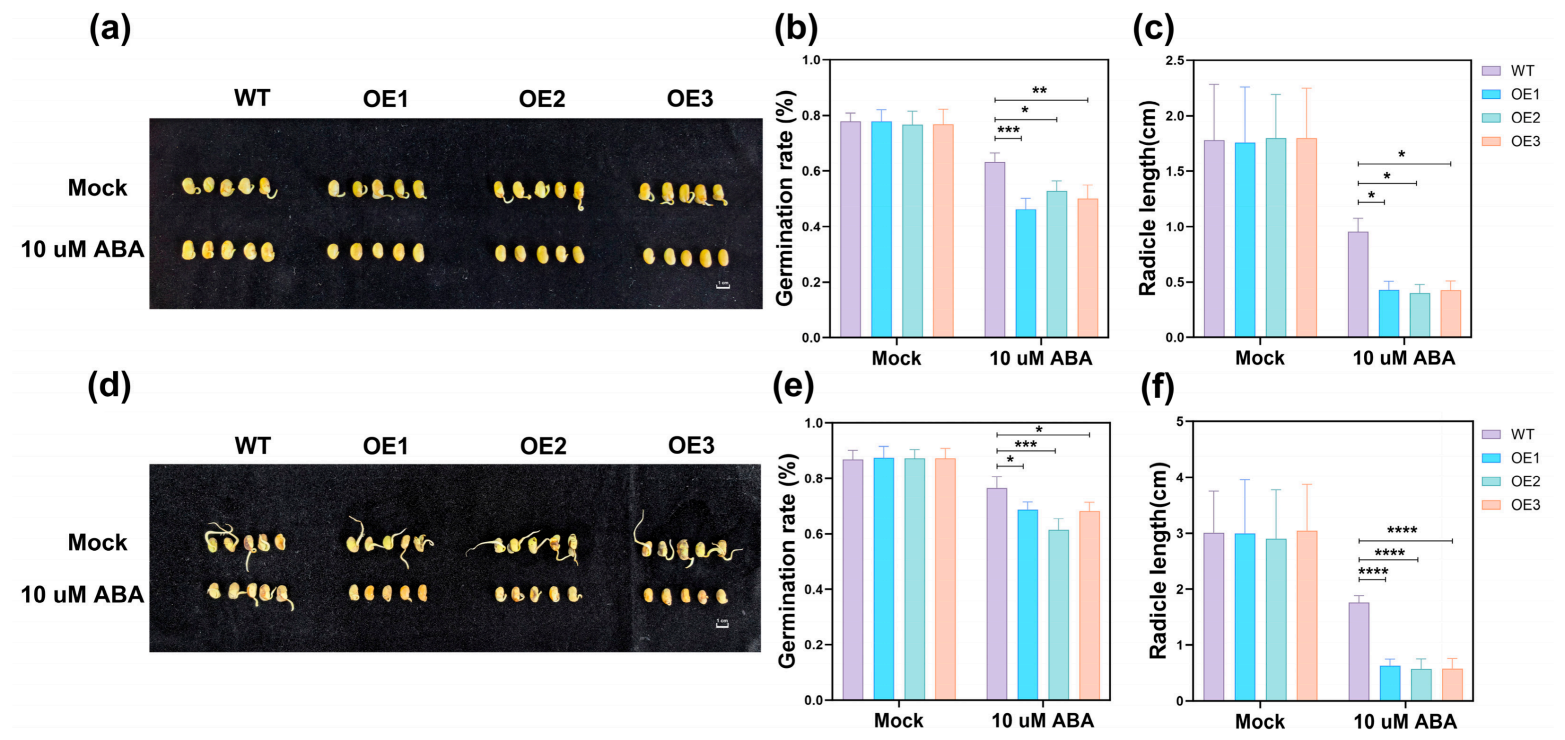
Effects of ABA treatment on seed germination of wild-type (WT) and transgenic soybeans (OE1, OE2, OE3). Seeds were germinated under Mock and 10 μM ABA treatments. (**a**–**c**) Germination phenotype, germination potential and radicle length at 4 d; (**d**–**f**) Germination phenotype, germination rate and radicle length at 7 d. Data represent the mean ± standard error (Mean ± SE, *n* = 3) from three independent biological replicates. Asterisks indicate significant differences (Student’s *t*-test), * *p* < 0.05, ** *p* < 0.01, *** *p* < 0.001, **** *p* < 0.0001. Scale bar = 1 cm.

## Data Availability

The original contributions presented in the study are included in the article/Supplementary Materials.

## Supplementary Materials

The following supporting information can be downloaded at https://www.mdpi.com/article/10.3390/plants15192939/s1: Figure S1: The CDS and deduced amino acid sequences of *AcCHI* from *Atriplex canescens*; Figure S2: Identification of transgenic plants; Table S1: Primers used in this study for the identification of screening markers in soybeans; Table S2: Primers used in this study for PCR; Table S3: Primers used in this study for qRT-PCR.
