# Supplementary material for: AcCHI, a Chitinase from Atriplex canescens, Confers Enhanced Tolerance to Osmotic and Salt Stress in Glycine max by Reinforcing Antioxidant and Osmotic Responses
Source: Plants (Basel). 2026 Sep 25;15(19):2939. doi: 10.3390/plants15192939 (PMC13666615; doi:10.3390/plants15192939)
Supplement: Supplementary file 1 [file plants-15-02939-s001.zip › plants-4463736-supplementary.pdf]

Supplementary Materials

>AcCHI
ATGTCTTCTATTAGACAATTTTTCTCCATACTAATGGCCGTTGCTTGTATGTCATGCGCCGCCCTTGTTGCG
GCTCAAAACTGTGGTTGTGTTTCTAACTTTTGCTGTAGCAAATATGGTTACTGTGGGACCACAAAAGCCTAT
TGTGGTGACGGATGCCAAAGTGGCCCTTGTTACTCTAAAACCCCGACAACCCCATCAGGTGGTGGTGGCG
GTGGTGCTGGTGCTGCCTCTGTCCGAAGTTTGGTGACGGACTCGTTCTTTAATGGGATCAAAAACCAAGCA
GGCTCTGGCTGTGCTGGTAAGAGCTTCTACACCAGATCTGCTTTCTTGAACGCTCTCAATGGCTATTCTAAG
TTCGGGACTGGTGGTTCCTCCGATGATACTAAGCGTGAAGTTGCCGCCTTCTTTGCTCATGTCACCCATGAAA
CTGGACATTTTTGCTACATAGAGGAGATTGCGAAATCAACCTACTGTCAATCAAGTGCACAATGGCCAT
GCAACCCAAACAAGCAATACTACGGTAGAGGACCAATTCAGATAACATGGAACTACAACTACGAAGCA
GCGGGTAAAAGCATTGGGTTTGATGGTATAAATGCACCAGAAACAGTTGCCAACAATGCAGTAATAGCC
TTCAAGACAGCTTTCTGGTTTTGGATGAACAATGTCCACTCTAAGATCATATCCGGCCAAGGGTTCGGGTC
CACCATTCGCGCTGTCAATGGCGGCGAATGTGGTGGTGGAAATACTGCTGCTGTCAATGCTCGTGTTGGG
TACTATACTCAGTACTGTAAGCAGTTAGGTGTTTCTCCTGGAAACAACCTTTCTTGCTAA

MSSIRQFFSILMAVACMSCAALVAAQNCGCVSNFCCSKYGYCGTTKAYCGDGCQSGPCYSKTPTTPSGG
GGGGAGAASVRSLVTDSFFNGIKNQAGSGCAGKSFYTRSAFLNALNGYSKFGTGGSSDDTKREVAAFFAH
VTHETGHFCYIEEIAKSTYCQSSAQWPCNPNKQYYGRGPIQITWNYNYEAAGKSIGFDGINAPETVANNA
VIAFKTAFWFWMNNVHSKIISGQGFGSTIRAVNGGECGGGNTAAVNARVGYYTQYCKQLGVSPGNNLSC

Figure S1. The CDS and amino acid sequences of *AcCHI* from *Atriplex canescens*.

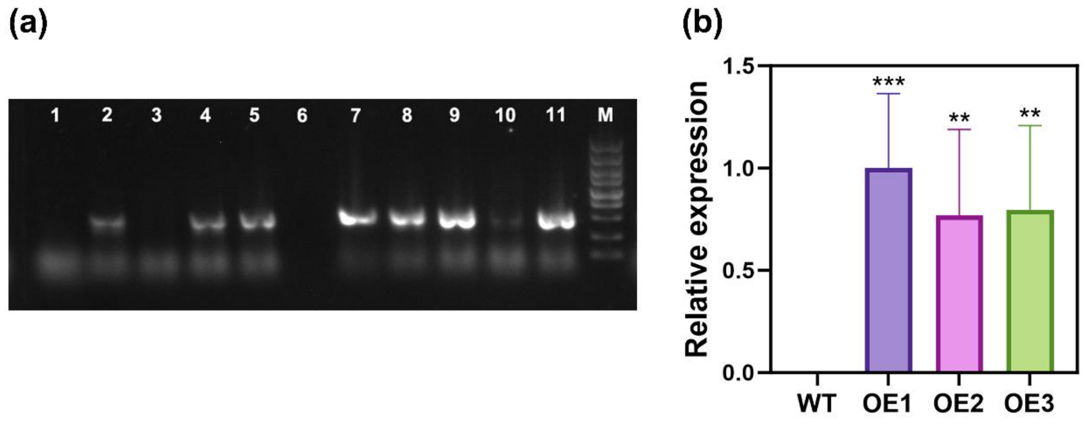

Figure S2. Identification of transgenic plants. (a) PCR-based identification of genomic integration in T_1-generation transgenic plants; Lane 1: wild-type "Shennong No. 9" (negative control); Lanes 2–11: regenerated plants obtained via Agrobacterium transformation; Lane M: DL5000 DNA Marker. The three lines with the brightest bands and the best amplification results (lanes 7, 8, and 9) were selected and named OE1, OE2, and OE3. (b) Relative transcript abundance of *AcCHI* in wild-type soybean and three independent overexpression lines (OE-1, OE-2, OE-3). Expression was normalized to *GmActin*. OE-1 was used as the calibrator for relative quantification among transgenic lines. No *AcCHI* transcript was detected in wild-type plants. Data represent mean ± SE of three biological replicates.

Table S1. Primers used in this study for the identification of screening markers in soybeans

Primer name	Primer Sequence (5′-3′)
bar-F	GCGGTCTGCACCATCGTCAACC
bar-R	CCAGAAACCCACGTCATGCCAGTT

Table S2. Primers used in this study for PCR

Primer name	Primer Sequence (5′-3′)
AcCHI(ORF)-F SacI	ccGAGCTCATGTCTTCTATTAGACAATTTTTCTCCA
AcCHI(ORF)-R BamHI	cgGGATCCTTAGCAAGAAAGGTTGTTTCCAG

Table S3. Primers used in this study for qRT-PCR

Primer name	Primer Sequence (5′-3′)
AcCHI-qRT-F	CTGGTGGTTCCTCCGATGATAC
AcCHI-qRT-R	TTACCCGCTGCTTCGTAGTTGT
GmDREB1B;1-qRT-F	GTAAAGATTGTTCGTATGGGACAAG
GmDREB1B;1-qRT-R	ACACCTAAAATGAGCAACCGTACTA
GmRD22-qRT-F	GGAGTATCGTCTCCTACCCAT
GmRD22-qRT-R	GTTGACATTGGTGCCACCTC
GmDREB2-qRT-F	ACCCAACAACAGGGCAAGAT
GmDREB2-qRT-R	GCTGACGTGGCTGGAACTAA
GmSOS1-qRT-F	CACTGCTCAGGAGGGTTCAG
GmSOS1-qRT-R	GAGAAGCAAGAGGTGGGTCC
GmNHX1-qRT-F	GTCGGGGCACACTTCACTAA
GmNHX1-qRT-R	GGATGCTGCTTGGACGATGA
GmCLC1-qRT-F	TTGTGGTGCTTCTTCAGGGG
GmCLC1-qRT-R	AAGGCTTCCCAATACCCCAC
Actin11-qRT-F	ATCTTGACTGAGCGTGGTTATTCC
Actin11-qRT-R	GCTGGTCCTGGCTGTCTCC
